# “Dealing with the Hospital has Become too Difficult for Us to Do Alone” – Developing an Integrated Care Program for Children with Medical Complexity (CMC)

**DOI:** 10.5334/ijic.3953

**Published:** 2018-09-05

**Authors:** Lisa Altman, Christie Breen, Joanne Ging, Sara Burrett, Tim Hoffmann, Emma Dickins, Kristen Brown, Yvonne Zurynski, Susan Woolfenden

**Affiliations:** 1 Sydney Children’s Hospitals Network, AU; 2 Murrumbidgee Local Health District, AU; 3 Macquarie University, AU; 4 University of Sydney, AU; 5 University of New South Wales, AU

**Keywords:** Children with medical complexity (CMC), care coordination, paediatric integrated care

## Abstract

**Introduction:**

Children with medical complexity (CMC) require highly specialised care, often from multiple providers and over many years. This paper describes the first 18 months of development of the Kids Guided Personalised Services (GPS) Integrated Care Program (the Program). This Program aims to improve health care experience; communication and to streamline provision of care.

**Discussion:**

Key enablers across the Program were put in place and 5 individual project streams were used to implement change. An extensive formative evaluation process was undertaken to truly understand all perspectives in developing the Program.

**Conclusion/Key Lessons:**

This Program supports families who are caring for CMC by developing shared care models that bring together local health services with the tertiary hospitals. The methodology used has resulted in comprehensive system change and transformation; reduced presentations to the Emergency Department (ED), avoidable admissions and travel time. A challenge remains in meaningfully engaging primary health care providers.

## Introduction

Children with medical complexity (CMC) have high levels of family identified health care needs, significant functional limitations and high health care utilisation [1,2]. The Australian health system is primarily designed for episodic care and does not adequately support children and families who deal with the on-going medical, emotional and practical impacts of managing a chronic condition. This can result in poor health outcomes, including unplanned hospital admissions, emergency department (ED) presentations, and longer hospitalisations. In the USA, fragmented health care is reportedly 35% more costly than integrated care, and this is thought to be similar in Australia [3].

An integrated care approach enables patient centred care across the health spectrum, bringing care closer to home and community, and empowers patients and families to manage their own care journey [4,5,6]. In 2015, Sydney Children’s Hospitals Network (SCHN) secured funding from the NSW Health Integrated Care Planning and Innovation Fund [7] to develop an integrated care model for CMC that reduced avoidable tertiary hospital encounters, and improved health outcomes, continuity of care, and family capacity to navigate the health system.

### Kids Guided Personalised Service (Kids GPS) Integrated Care Program

**Figure 1:**
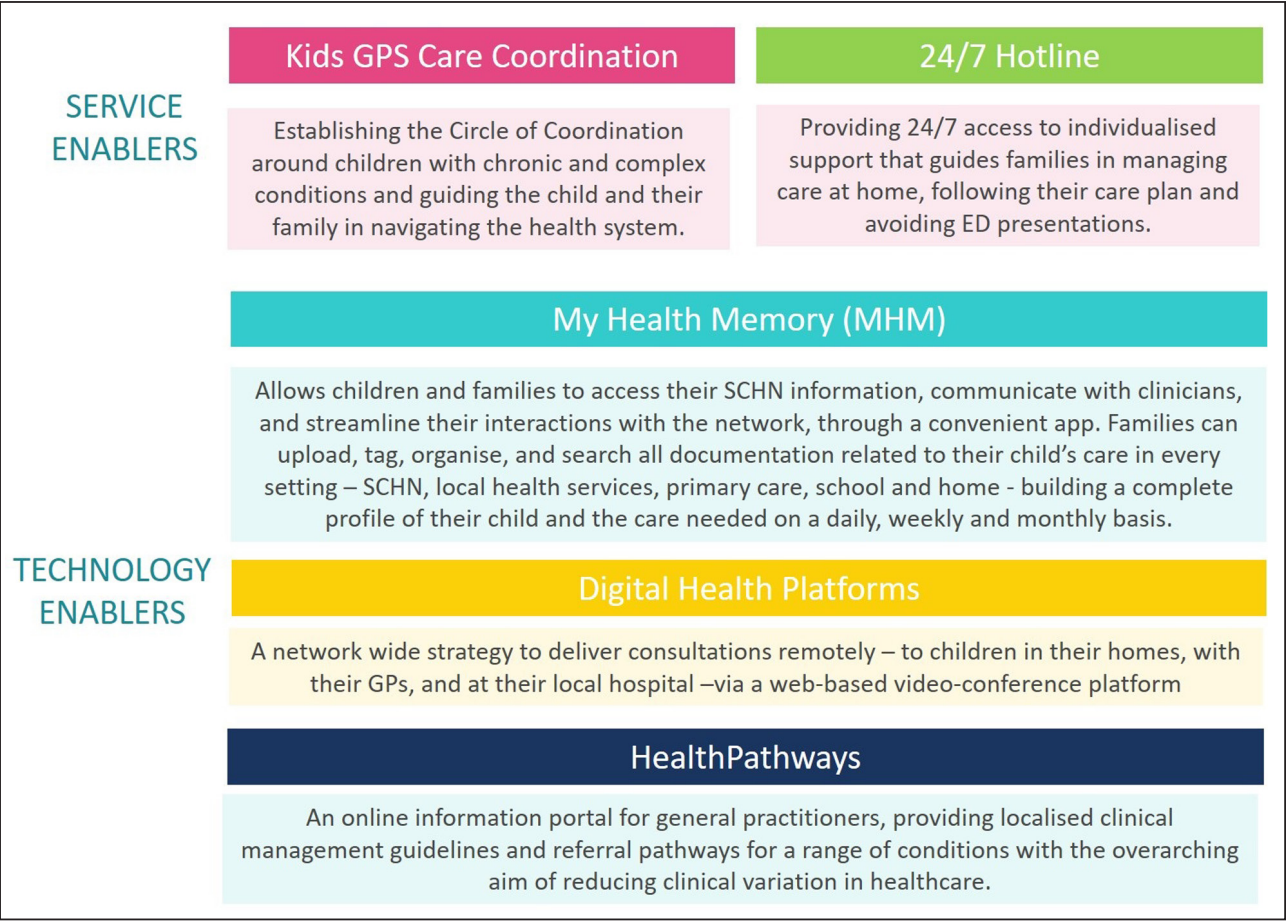
Service and technology enablers.

The SCHN Kids GPS Integrated Care Program (the Program) is based on extensive formative evaluation involving families, health care providers and system partners who called for key service and technology enablers (Figure 1). Service change is implemented through quality improvement (QI) projects and integrated systems redesign.

### Service Enablers

#### Kids GPS Care Coordination

**Figure 2:**
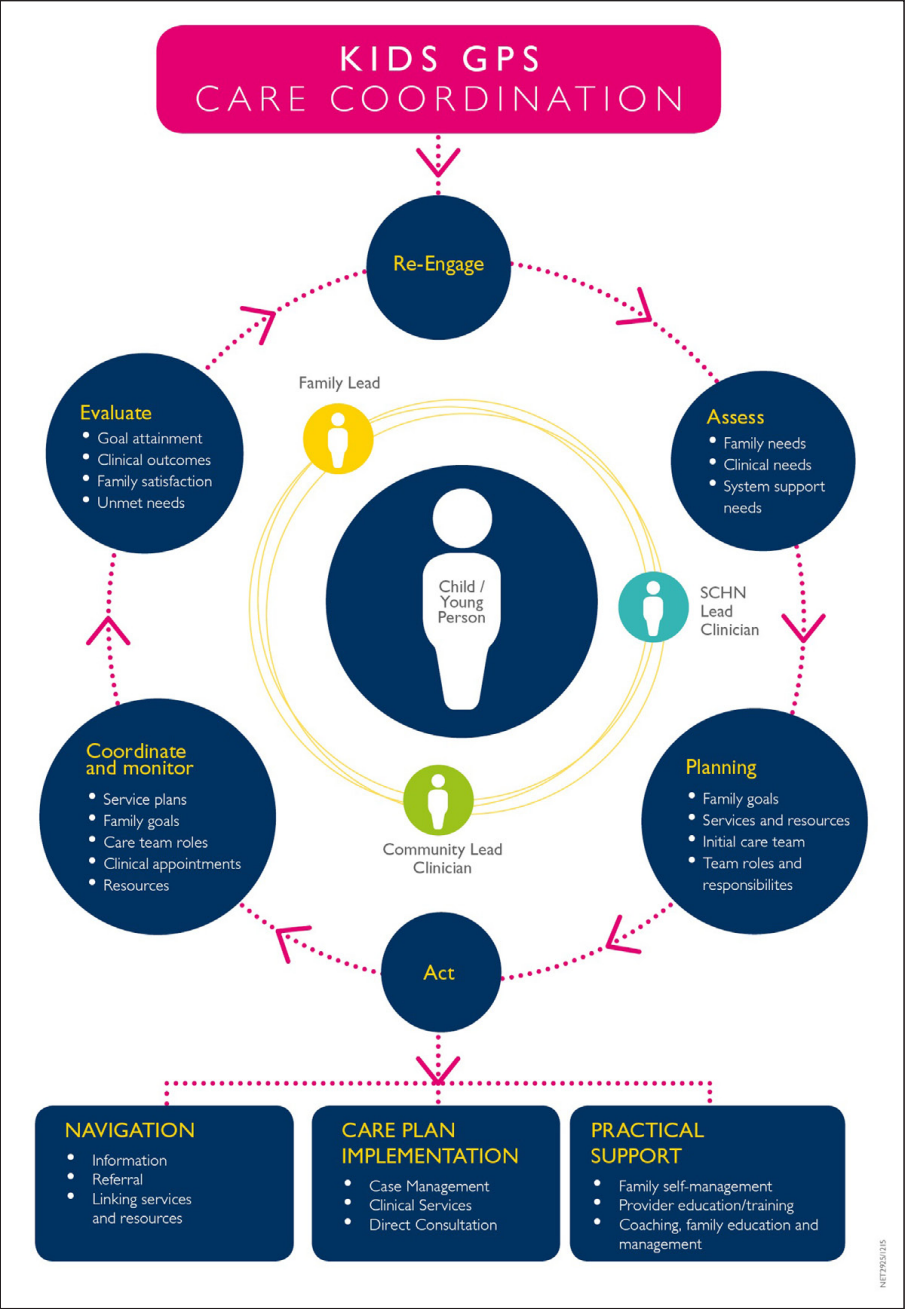
Kids GPS circle of care coordination. *Source:* Adapted from Cohen et al. [8].

Care coordination for CMC is a key foundation of the Program. We modified the model of integrated care described by Cohen et al. [8], where Care Coordinators are responsible for establishing the **Circle of Coordination** around CMC (Figure 2). Eligibility for care coordination is defined by medical complexity, frequency of hospital utilisation and the family’s psychosocial complexity. Identified ‘lead’ people within SCHN, the local community and the family share responsibility for meeting the health care needs of the child. Care Coordinators work with all parties to facilitate communication, reinforced through shared care plans developed jointly with the family and the leads. Shared care plans include medical history, diagnoses, current needs, medications, treatment goals, and service providers. In the first two years of the service 534 CMC were enrolled in the care coordination service. A 24/7 Hotline enables families of children who are medically fragile to access support at any time, empowering them to gain confidence in managing their child’s condition at home. Families of children presenting to ED frequently, are encouraged to call the hotline first for guidance on whether the child needs to attend the tertiary hospital, a local hospital, or community-based service. Fifty-five families have accessed the hotline.

### Technological Enablers

#### My Health Memory

**Figure 3:**
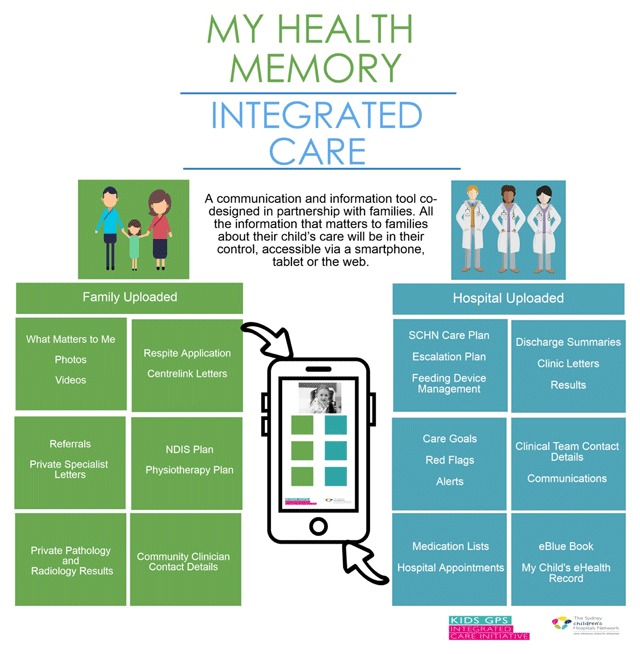
My Health Memory app components.

The formative evaluation identified the need for better systems of communication among health services and families. Families were frustrated at repeating their story to multiple clinicians and frequently carried a ‘manual’ containing medical letters, business cards, results, reports and lists of appointments. The My Health Memory (MHM) smart-phone app co-designed with families (Figure 3), provides a convenient way to access and share their child’s SCHN information. The shared care plan is ‘pushed’ from the SCHN Electronic Medical Record to MHM in real time. Families can also upload, tag, organise, search and store all documentation related to care in every health care setting. MHM is continually evaluated, with 3627 families currently actively using it.

#### Digital Health Platforms

Families caring for CMC, experience logistical challenges when accessing health services, often requiring time off work and school, additional care for siblings, and long waiting times in clinic. Transporting mobility restricted children or those who require respiratory support is also difficult. Families from regional/rural areas have worse experiences and incur additional costs for travel and accommodation. SCHN has implemented Telehealth via a web-based video-consultation platform for remote consultation delivery – to children at home, with general practitioners (GPs) and at local hospitals.

#### HealthPathways

HealthPathways is an online information portal for GPs that provides localised clinical management and referral pathways. Currently there are over 80 paediatric pathways in Western Sydney [9]. During 2017, the top five most accessed paediatric pathways were: Developmental Milestones, Developmental Concerns in Children, Urinary Tract Infection in Children, ADHD in Children and Youth, and Weight Management in Children and Adolescents.

### Implementing systems change

**Figure 4:**
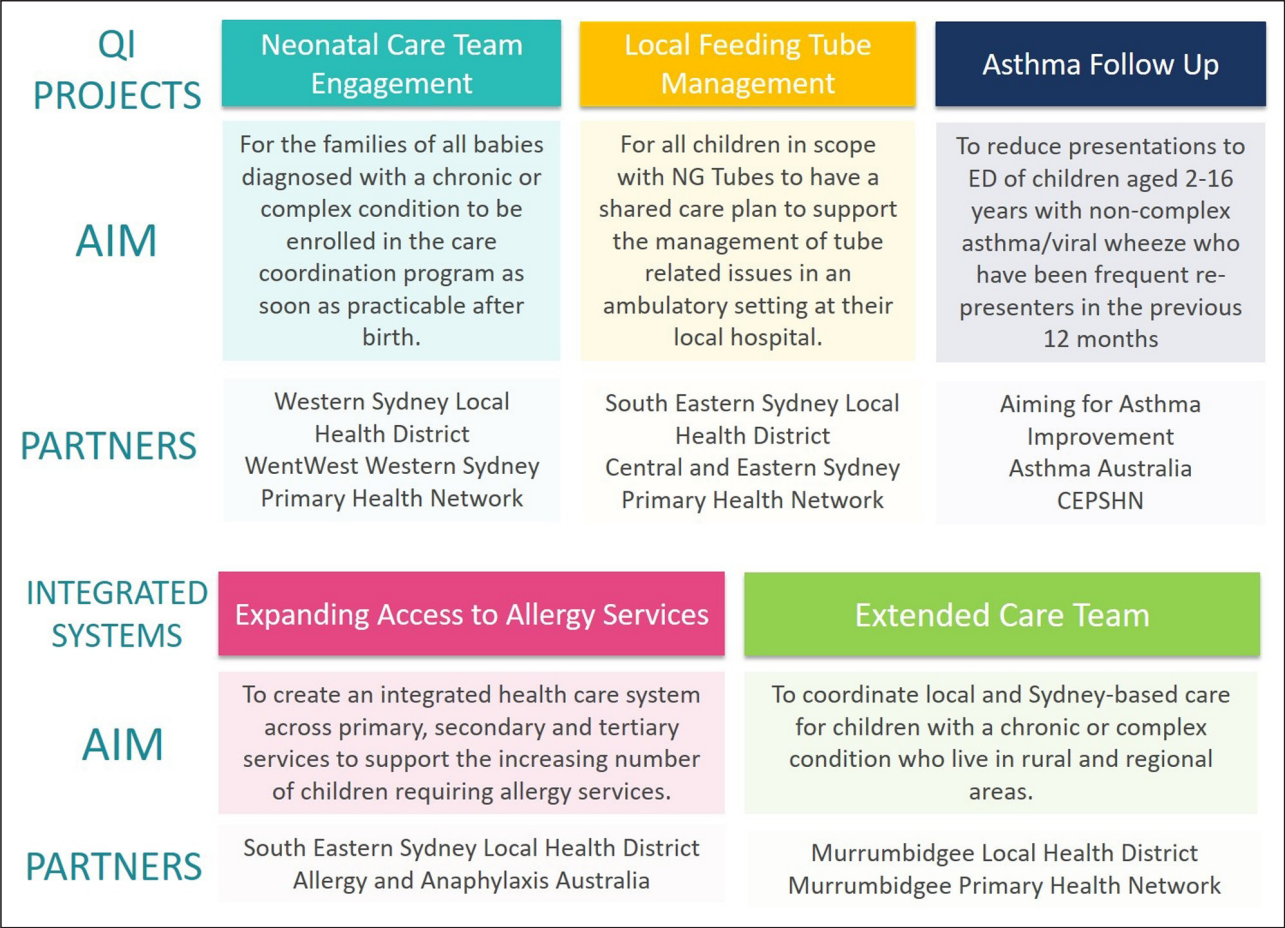
Quality improvement projects and integrated systems redesign projects to support health care systems change.

Brokering partnerships across multiple organisations and facilitating change was supported by using QI methodology and integrated systems co-redesign. Three QI projects for targeted cohorts: – Asthma Follow-up, South Eastern Sydney Local Replacement of Nutrition Support Devices, and Western Sydney Neonatal Care Team Engagement – were established to pilot-test impact of linking tertiary and local hospitals, and primary care. Two integrated system redesign projects focused on streamlining management of paediatric allergy, and improving services for CMC in rural areas, (Figure 4). Over 45 care professionals (sub-specialists, paediatricians, nurses, GPs, Allied Health practitioners) at SCHN and in the community have partnered with the Program to implement the QI projects. Examples of successful outcomes include: 24 infants likely to have medical complexity were identified at birth and enrolled; asthma presentations to ED have halved for children with recurrent presentations (to be published elsewhere).

### Methods for Evaluation and Summary of findings

Formative evaluation activities undertaken in 2015/2016 as a foundation for the Program co-design included stakeholder forums and interviews, interviews with parents of CMC, and analysis of baseline CMC data. To support the design of MHM an observational study “Patient Shadowing” project was undertaken.

**Figure 5:**
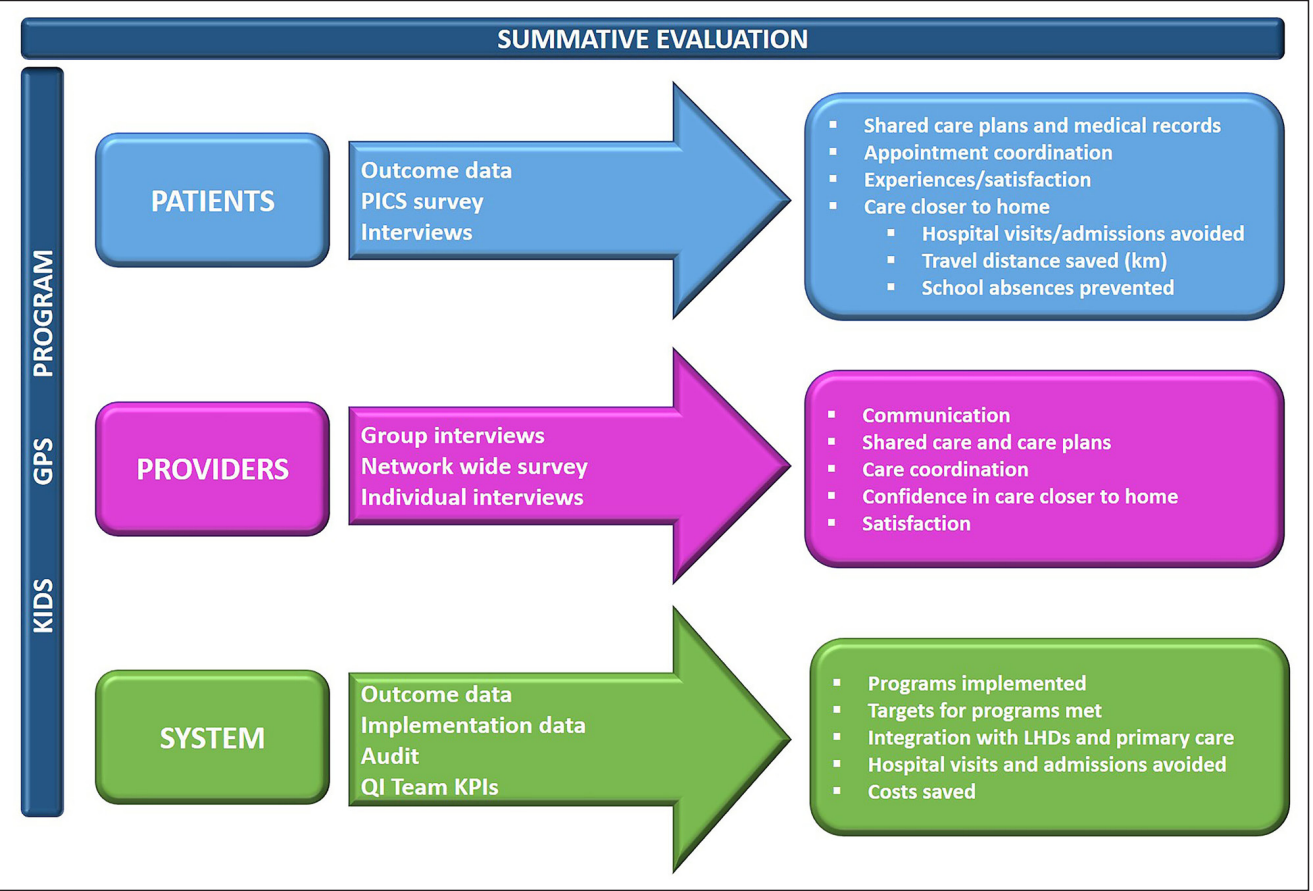
Summative evaluation. PISC: Pediatric Integrated Care Survey. KPIs: Key Performance Indicators. QI: Quality Improvement.

A mixed methods approach underpinned the summative evaluation activities which aimed to evaluate impact at the patient, provider and system level, (Figure 5). The methods included pre- and post- implementation analysis of hospital administrative data on encounters, surveys of parents/carers using the Pediatric Integrated Care Survey [10] to survey SCHN providers (doctors, nurses and allied health); and interviews with QI teams and Care Coordinators (Figure 5). The summative evaluation findings will be published in greater detail elsewhere. In summary, analysis of hospital encounters 6 months pre- and post- enrolment in care coordination found a reduction in ED presentations by 40%; day only admissions by 42% and savings of over 50,000 kilometres of travel for families of CMC.

## Reflection

The development of paediatric integrated care models in Australia is new and peer reviewed literature reporting outcomes of such models outside of North America is lacking [4,11,12,13]. We were aware of multiple paediatric integrated care services across Australia, but there is only one published study showing clear benefits of care coordination and a 24-hour phone line [5]. Our preliminary data shows a clear reduction in ED presentations, hospital admissions and travel time. At least another three papers on the outcomes of this Program are planned.

Care Coordinators are central to the successful and enduring implementation of this Program. Experienced and highly skilled paediatric nurses were employed as Care Coordinators as the role requires them to provide much needed support to CMC and their healthcare teams, and to lead culture change needed for successful implementation of the Program. The Program project team used QI methodology to overcome entrenched traditional episodic ways of providing care which posed barriers to referral of CMC to the Care Coordinators. Thus, key internal and external contextual factors to the success of care coordination were addressed. This is in line with the recommendations of a recent systematic review on the role of care coordinators [14].

Successful integration needs a multidisciplinary whole of system approach, with deep engagement of families and stakeholders, equitable reach, and evidence-based standardised care. Service delivery reform needs to be driven by QI methodology. Information systems are needed to ensure optimal communication across the health system [15]. We included all of these components in our Program design. The support of a state-wide policy to drive integration, coupled with political support and increasing investment in workforce planning, has been invaluable [7,15].

A number of challenges remain. Although we have partner Primary Health Networks and consulted widely with GPs, we have only recently begun to engage with GPs and Practice Nurses in implementing the Program. We first needed to provide clear pathways for CMC across SCHN and the LHDs. The funding available for the Integrated Care program was initially annual project funding from the NSW Health Planning and Innovation Fund which had an impact on the capacity for long-term planning. However NSW Health is now providing recurrent funding for the Program at SCHN.

## Conclusion

Through this Program we have established a safety net for CMCs as they navigate our health system. By incorporating evaluation activities from the outset, we have demonstrated a beneficial impact for CMC and their families and generated much needed evidence to inform future paediatric integrated care models. A priority for the next 5 years of this service is to develop meaningful partnerships with GPs to develop local solutions that support them in managing CMC.

## Author Information

The Sydney Children’s Hospitals Network (SCHN) is the largest paediatric health entity in Australia, with a team of 5,000 staff committed to providing world-class paediatric health care in a family-focussed, healing environment. Sydney Children’s Hospitals Network provides care for over 120,000 families each year, incorporating 50,000 inpatient admissions, 90,000 emergency presentations and over 1 million outpatient services.
